# Opportunities and Challenges of Cloud Computing to Improve Health Care Services

**DOI:** 10.2196/jmir.1867

**Published:** 2011-09-21

**Authors:** Alex Mu-Hsing Kuo

**Affiliations:** ^1^School of Health Information ScienceUniversity of VictoriaVictoria, BCCanada

**Keywords:** Health care, electronic health record, cloud computing, bioinformatics, quality improvement

## Abstract

Cloud computing is a new way of delivering computing resources and services. Many managers and experts believe that it can improve health care services, benefit health care research, and change the face of health information technology. However, as with any innovation, cloud computing should be rigorously evaluated before its widespread adoption. This paper discusses the concept and its current place in health care, and uses 4 aspects (management, technology, security, and legal) to evaluate the opportunities and challenges of this computing model. Strategic planning that could be used by a health organization to determine its direction, strategy, and resource allocation when it has decided to migrate from traditional to cloud-based health services is also discussed.

## Introduction

Cloud computing refers to an on-demand, self-service Internet infrastructure that enables the user to access computing resources anytime from anywhere [1]. It is a new model of delivering computing resources, not a new technology. Examples of commonly used non-health care applications include Microsoft Hotmail and Google Docs, while some better known applications in health care include Microsoft HealthVault and Google Health platform (recently discontinued [2]). However, compared with conventional computing, this model provides three new advantages: massive computing resources available on demand, elimination of an up-front commitment by users, and payment for use on a short-term basis as needed [3]. Several articles, forums, and blogs have reported its applications in industry, business, transportation, education, and national security [4-7].

Health care, as with any other service operation, requires continuous and systematic innovation in order to remain cost effective, efficient, and timely, and to provide high-quality services. Many managers and experts predict that cloud computing can improve health care services, benefit health care research, and change the face of information technology (IT) [8-13]. For example, Schweitzer [10], Haughton [11], and Kabachinski [12] believe that cloud computing can reduce electronic health record (EHR) startup expenses, such as hardware, software, networking, personnel, and licensing fees, and therefore will encourage its adoption. Research by Rosenthal et al shows that the biomedical informatics community, especially consortiums that share data and applications, can take advantage of the new computing paradigm [13]. As indicated in the paper by Anderson et al, data-handling problems, complexity, and expensive or unavailable computational solutions to research problems are major issues in biomedical research data management and analysis [14]. Several informatics innovations have demonstrated that cloud computing has the potential to overcome these difficulties [15-21].

Despite the many benefits associated with cloud computing applications for health care, there are also several management, technology, security, and legal issues to be addressed. The aim of this paper is to discuss the concept of cloud computing, its current applications in health care, the challenges and opportunities, and how to implement strategic planning when the organization has decided to move to the new model of service.

## Cloud Computing: A New Economic Computing Model

Cloud computing is still a developing paradigm, and its definition, attributes, and characteristics will evolve over time. Vaquero et al studied more than 20 definitions and tried to extract a consensus definition as well as a minimum definition containing the essential characteristics. Based on the study, they defined cloud computing as follows [22]:

Clouds are a large pool of easily usable and accessible virtualized resources (such as hardware, development platforms and/or services). These resources can be dynamically re-configured to adjust to a variable load (scale), allowing also for an optimum resource utilization. This pool of resources is typically exploited by a pay-per-use model in which guarantees are offered by the Infrastructure Provider by means of customizedService-Level Agreements.

From a service point of view, cloud computing includes 3 archetypal models: software, platform, and infrastructure [1,23-25]. 

(1) *Software as a service (SaaS):* The applications (eg, EHRs) are hosted by a cloud service provider and made available to customers over a network, typically the Internet.

(2) *Platform as a service (PaaS):* The development tools (eg, operation systems) are hosted in the cloud and accessed through a browser. With PaaS, developers can build Web applications without installing any tools on their computer, and then deploy those applications without any specialized administrative skills.

(3) *Infrastructure as a service (IaaS):* The cloud user outsources the equipment used to support operations, including storage, hardware, servers, and networking components. The provider owns the equipment and is responsible for housing, running, and maintaining it. The user typically pays on a per-use basis.

**Figure 1:**
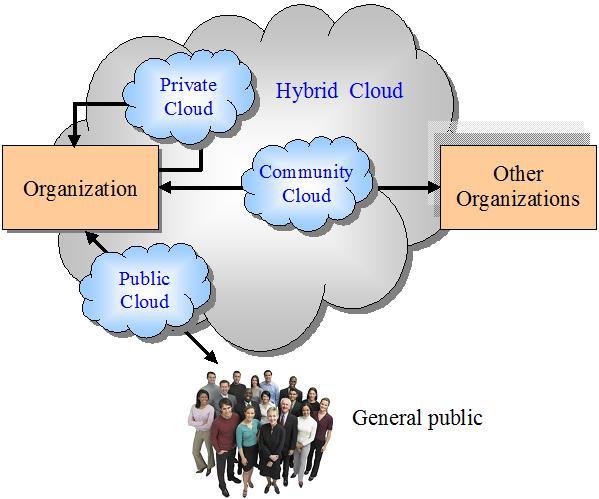
The cloud computing deployment models.

To deploy cloud computing, the US National Institute of Standards and Technology (NIST) listed 4 models (see Figure 1) [1,26]:

(1) *Public cloud:* A cloud service provider makes resources (applications and storage) available to the general public over the Internet on a pay-as-you-go basis. For example, the Amazon Elastic Compute Cloud (EC2) allows users to rent virtual computers on which to run their own applications. EC2 runs within Amazon’s network infrastructure and data centers and allows customers to pay only for what they use with no minimum fee.

(2) *Private cloud:* A cloud infrastructure is operated solely for a single organization. In other words, the proprietary network or the data center supplies hosted services to a certain group of people. For example, Microsoft Azure enables customers to build the foundation for a private cloud infrastructure using Windows Server and System Center family of products with the Dynamic Data Center Toolkit. 

(3) *Community cloud:* The cloud infrastructure is shared by several organizations with common concerns (eg, mission, security requirements, policy, and compliance considerations). For example, the Google GovCloud provides the Los Angeles City Council with a segregated data environment to store its applications and data that are accessible only to the city’s agencies.

(4) *Hybrid cloud:* The cloud infrastructure comprises 2 or more clouds (private, public, or community). In this infrastructure, an organization provides and manages some resources within its own data center and has others provided externally. For example, IBM collaborates with Juniper Networks to provide a hybrid cloud infrastructure to enterprises to seamlessly extend their private clouds to remote servers in a secure public cloud [27].

## Status and Adoption of Cloud Computing in Health Care

Many previous studies reported the potential benefits of cloud computing and proposed different models or frameworks in an attempt to improve health care service [28-35]. Among them, Rolim et al proposed a cloud-based system to automate the process of collecting patients’ vital data via a network of sensors connected to legacy medical devices, and to deliver the data to a medical center’s “cloud” for storage, processing, and distribution. The main benefits of the system are that it provides users with 7-days-a-week, real-time data collecting, eliminates manual collection work and the possibility of typing errors, and eases the deployment process [36]. Nkosi and Mekuria described a cloud computing protocol management system that provides multimedia sensor signal processing and security as a service to mobile devices. The system has relieved mobile devices from executing heavier multimedia and security algorithms in delivering mobile health services. This will improve the utilization of the ubiquitous mobile device for societal services and promote health service delivery to marginalized rural communities [37]. Rao et al reported a pervasive cloud initiative called Dhatri, which leveraged the power of cloud computing and wireless technologies to enable physicians to access patient health information at anytime from anywhere [38]. Koufi et al described a cloud-based prototype emergency medical system for the Greek National Health Service integrating the emergency system with personal health record systems to provide physicians with easy and immediate access to patient data from anywhere and via almost any computing device while containing costs [39].

Numerous of articles and resources also reported the successful application of cloud computing in bioinformatics research [15-21,40,41]. For example, Avila-Garcia et al proposed a framework based on the cloud computing concept for colorectal cancer imaging analysis and research for clinical use [18]. Bateman and Wood used Amazon’s EC2 service with 100 nodes to assemble a full human genome with 140 million individual reads requiring alignment using a sequence search and alignment by hashing (SSAHA) algorithm [19]. Kudtarkar et al also used Amazon’s EC2 to compute orthologous relationships for 245,323 genome-to-genome comparisons. The computation took just over 200 hours and cost US $8,000, approximately 40% less than expected [20]. Memom et al applied cloud computing to evaluate the impact of G-quadruplexes on Affymetrix arrays [21]. The Laboratory for Personalized Medicine of the Center for Biomedical Informatics at Harvard Medical School took the benefits of cloud computing to develop genetic testing models that managed to manipulate enormous amounts of data in record time [41].

Besides academic researchers, many world-class software companies have heavily invested in the cloud, extending their new offerings for medical records services, such as Microsoft’s HealthVault, Oracle’s Exalogic Elastic Cloud, and Amazon Web Services (AWS), promising an explosion in the storage of personal health information online. Also, the use of health cloud computing is reported worldwide. For example, the AWS plays host to a collection of health care IT offerings, such as Salt Lake City-based Spearstone’s health care data storage application, and DiskAgent uses Amazon Simple Storage Service (Amazon S3) as its scalable storage infrastructure [42].

The American Occupational Network is improving patient care by digitizing health records and updating its clinical processes using cloud-based software from IBM Business Partners MedTrak Systems. The company now can provide faster and more accurate billing to individuals and insurance companies, shortening the average time to create a bill from 7 days to less than 24 hours, and reducing medical transcription costs by 80% [43].

The US Department of Health & Human Services’ Office of the National Coordinator for Health Information Technology recently chose Acumen Solutions’ cloud-based customer relationship management and project management system for the selection and implementation of EHR systems across the United States. The software enables regional extension centers to manage interactions with medical providers related to the selection and implementation of an EHR system [44].

Telstra and the Royal Australian College of General Practitioners announced the signing of an agreement to work together to build an eHealth cloud. Telstra is one of the leading telecommunications providers in Australia; the College is the largest general practice representative body in Australia with more than 20,000 members and over 7000 in its National Rural Faculty. The eHealth cloud will host health care applications including clinical software, decision-support tools for diagnosis and management, care plans, referral tools, prescriptions, training, and other administrative and clinical services [45].

In Europe, a consortium including IBM, Sirrix AG security technologies, Portuguese energy and solution providers Energias de Portugal and EFACEC, San Raffaele Hospital (Italy), and several European academic and corporate research organizations contracted Trustworthy Clouds—a patient-centered home health care service—to remotely monitor, diagnose, and assist patients outside of a hospital setting. The complete lifecycle, from prescription to delivery to intake to reimbursement, will be stored in the cloud and will be accessible to patients, doctors, and pharmacy staff [46].

## Health Cloud Computing Opportunities and Challenges

Recent research indicates that 75% of chief information officers reported that they will need and use cloud computing in the near future [47,48]. The forecast, conducted by Mark Beccue, suggested that the number of people subscribing to mobile cloud applications will rise from 71 million to nearly a billion by 2014 [49]. In health sectors, many organizations, managers, and experts believe that the cloud computing approach can also improve services and benefit research [8-13]. In addition, a report by the European Network and Information Security Agency (ENISA) stated that this new computing model is set to see massive global investment in many sectors, including health care [50]. The report also estimated that, by 2013, US $44 billion will be spent worldwide on cloud computing, potentially providing huge benefits to health care.

As with any innovation, cloud computing should be rigorously evaluated before its widespread adoption. Few research papers have systematically studied the impact of cloud computing on health care IT in terms of its opportunities and challenges. This study reviews the literature and evaluates the opportunities and challenges from the viewpoint of management, technology, security, and legality (see Table 1).

**Table 1:** Cloud computing opportunity and challenge summary

Aspects	Opportunities	Challenges
Management	Lower cost of new IT^a^ infrastructure	Lack of trust by health care professionals
	Computing resources available on demand	Organizational inertia
	Payment of use on a short-term basis as needed	Loss of governance
		Uncertain provider’s compliance
Technology	Reduction of IT^a^ maintenance burdens	Resource exhaustion issues
	Scalability and flexibility of infrastructure	Unpredictable performance
	Advantage for green computing	Data lock-in
		Data transfer bottlenecks
		Bugs in large-scale distributed cloud systems
Security	More resources available for data protection	Separation failure
	Replication of data in multiple locations increasing data security	Public management interface issues
	Dynamically scaled defensive resources strengthening resilience	Poor encryption key management
		Privilege abuse
Legal	Provider’s commitments to protect customer’s data and privacy	Data jurisdiction issues
	Development of guidelines and technologies to enable the construction of trusted platforms by not-for-profit organizations	Privacy issues
	Fostering of regulations by government for data and privacy protection	

^a^ Information technology.

### Management Aspect

#### Opportunity

The principle advantage of cloud computing is its low cost. For example, Amazon charges only US $0.1 per hour for 1.0-GHz × 86 instruction set architecture “slices” of EC2. Amazon S3 charges US $0.12 to $0.15 per gigabyte-month, with additional bandwidth charges of US $0.10 to $0.15 per gigabyte to move data into and out of AWS over the Internet [51]. An organization can easily get a cost-effective and on-premise IT solution through cloud computing without the need to purchase or evaluate hardware or software, or to hire internal IT staff to maintain and service in-house infrastructure [20,41,51]. The result is that the organization can focus on critical tasks without having to incur additional costs with regard to IT staffing and training.

Also, the cloud computing approach speeds deployment while maintaining vital flexibility (ie, rapid elasticity and ubiquitous access to health resources). This capability means that, as demand changes, hospitals and other health care providers do not need to adjust their infrastructures to accommodate the changes.

#### Challenges

The main challenges include lack of trust in data security and privacy by users, organizational inertia, loss of governance, and uncertain provider’s compliance.

Trust is at the heart of the resistance that many customers have to the cloud [52]. Concerns arise when their sensitive data and mission-critical applications move to a cloud computing paradigm where providers cannot guarantee the effectiveness of their security and privacy controls [53].

Cultural resistance (ie, organizational inertia) to share data and change traditional ways of working is a common management challenge to adopting cloud computing.

In some cases, a service level agreement may not offer a commitment to allow the client to audit its data. The loss of data governance could have a severe impact on a cloud user’s strategy and therefore on the capacity to meet its mission and goals.

Finally, if a provider cannot meet the requisite compliance norms (eg, applicable laws, regulations, standards, contracts, or policy changes), then a customer’s investment may be at risk. In some cases, certain customer services (eg, credit card transactions) cannot be used [54].

### Technology Aspect

#### Opportunity

Smaller hospitals, medical practices, and laboratories typically do not have internal IT staff to maintain and service in-house infrastructure for mission-critical applications such as EHRs. Therefore, eliminating the new infrastructure cost and the IT maintenance burdens can remove many obstacles to EHR adoption [10,55]. For bigger health organizations, placing data storage or IT application needs in the hands of a cloud provider essentially shifts the IT management burden to a third-party provider. From an IT management’s point of view, cloud computing can increase the scalability, flexibility, and cost effectiveness of infrastructure.

Also, cloud computing has advantages for so-called *green computing*—the more efficient use of computer resources to help the environment and promote energy saving. Usage of ready-made computing resources tailored to an organization’s needs certainly helps it to reduce electricity expenses. While it saves on electricity, it also saves on resources required to cool off computers and other components. This reduces the emission of dangerous materials into the environment [56].

#### Challenges

Several technical challenges related to the use of cloud computing include resource exhaustion, unpredictability of performance, data lock-in, data transfer bottlenecks, and bugs in large-scale distributed cloud systems.

Low cost and computing resources available on demand are two key features of cloud computing. However, the market is becoming crowded with large providers. Because of high competition, many cloud providers overcommit computing resources (eg, central processing unit [CPU] allocation, storage space, applications) to attract customers. In order to maintain the profit, they cut corners in the value-delivery system. For example, they may limit access to the cloud resources, or use out-of-date hardware or software or deploy older CPU technology. Unfortunately, most cloud customers are unable to govern the virtual architecture, and the providers usually do not permit an audit by the customers. The result is variable leading to unpredictable performance in the service [57]. This difference between the customer’s expectation and what the provider can really deliver presents a major technical challenge for the cloud customer to provide high-quality service to its own users.

Data lock-in is also an important challenge. In some cases, cloud users may have to move data or services to another provider or back to an in-house IT environment because the provider ceases business or service operations. For example, Google decided to discontinue its Google Health service on January 1, 2012. Users have a year to download their health data [2]. Unfortunately, most cloud infrastructures provide very little capability on data, application, and service interoperability [51]. This makes it difficult for a customer to migrate from one provider to another, or move data and services back to an in-house IT environment.

Some cloud users (eg, biomedical research laboratories) may need to frequently upload to or download very large amounts of data from the cloud. Application users may find that there is a data transfer bottleneck because of physical networking bandwidth limitation. Another specific technical risk is that of bugs in large-scale distributed cloud systems. When compared with in-house IT systems, the errors in these very large distributed infrastructures are more difficult to debug [51].

### Security Aspect

#### Opportunity

Perhaps the strongest resistance to the adoption of cloud computing in health IT centers relates to data security [58]. Nevertheless, compared with locally housed data, this model is not necessarily less secure. In some cases, it typically improves security because cloud providers (eg, Microsoft, Google, Amazon) are able to devote huge resources to solving security issues that many customers cannot afford, in contrast to the destruction of many medical records and legal documents in the Japan 9.0 magnitude earthquake or the New Orleans Hurricane Katrina disaster. 

All kinds of security measures, such as in hardware, software, human resources, and management costs, are cheaper when implemented on a large scale. Most cloud providers replicate users’ data in multiple locations. This increases data redundancy and independence from system failure and provides a level of disaster recovery. In addition, a cloud provider always has the ability to dynamically reallocate security resources for filtering, traffic shaping, or encryption in order to increase support for defensive measures (eg, against distributed denial-of-service attacks). The ability to dynamically scale defensive resources on demand has obvious advantages for resilience [54].

#### Challenges

There are many data security risks in the use of IT, such as hacker attacks, network breaks, natural disasters, separation failure, public management interface, poor encryption key management, and privilege abuse. Specific risks to cloud computing are separation failure, public management interface, poor encryption key management, and privilege abuse.

Cloud computing is usually accessible to many different customers. If the provider fails to separate the resources, it could cause very serious security risks. For example, a customer requests to delete data stored in the virtual infrastructure; as with most operating systems, this may not result in true erasing of the data immediately. The data are still stored on the disk but are just not available [54]. In the multiple tenancies environment, hardware resources are reused by other customers. In this case, a third party could have access to another customer’s “deleted” data. This presents a higher risk to the cloud customers than with dedicated hardware.

The public management interface is cloud computing’s other Achilles’ heel. As indicated in the ENISA’s cloud computing risk summary [54]:

The customer management interfaces of public cloud providers are Internet accessible and mediate access to larger sets of resources (than traditional hosting providers) and therefore pose an increased risk especially when combined with remote access and Web browser vulnerabilities

Strong encryption with key management is one of the core mechanisms that cloud computing systems use to guard against data loss and theft. However, a poor key management procedure may cause loss of encryption keys, disclosure of secret keys or passwords to malicious parties, or unauthorized use for authentication.

Lastly, as cloud use increases, employees may increasingly become targets for criminal organizations. If the malicious insider is a system administrator, then he or she could use his or her privileges to steal critical data.

### Legal Aspect

#### Opportunity

Data and privacy protection are essential to building the customer trust needed for cloud computing to reach its full potential. If the providers adopt better and clearer policies and practices, users would be better able to assess the related risks they face. Fortunately, many main providers have commitments to develop best policies and practices to protect customers’ data and privacy [59-61]. Besides providers’ commitments to this protection, some organizations, such as the Cloud Security Alliance, have developed a comprehensive guide to deal with security and privacy issues [62]. The Trusted Computing Group (http://www.trustedcomputinggroup.org/), a not-for-profit organization, suggests a set of hardware and software technologies to enable the construction of trusted platforms. Governments also play a critical role by fostering widespread agreement regulations for both users and providers [63-66].

#### Challenges

The use of cloud computing presents many legal issues such as contract law, intellectual property rights, data jurisdiction, and privacy [67-71]. Among them, data jurisdiction and privacy issues are major concerns.

In the cloud, physical storages could be widely distributed across multiple jurisdictions, each of which may have different laws regarding data security, privacy, usage, and intellectual property [70,71]. For example, the US Health Insurance Portability and Accountability Act (HIPAA) [63] restricts companies from disclosing personal health data to nonaffiliated third parties, and the Uniting and Strengthening America by Providing Appropriate Tools Required to Intercept and Obstruct Terrorism (PATRIOT) Act [72] gives the US government the right to demand data if it declares conditions as being an emergency or necessary to homeland security. Similarly, the Canadian Personal Information Protection and Electronic Documents Act (PIPEDA) [64] limits the powers of organizations to collect, use, or disclose personal information in the course of commercial activities. However, a provider may, without notice to a user, move the user’s information from jurisdiction to jurisdiction. Data in the cloud may have more than one legal location at the same time, with differing legal consequences.

Cloud computing is a shared resource and multitenancy environment for capacity, storage, and network. The privacy risk of this type of environment includes the failure of mechanisms for separating storage, memory, routing, and even reputation between different tenants of the shared infrastructure. The centralized storage and shared tenancy of physical storage space means the cloud users are at higher risk of disclosure of their sensitive data (eg, health records) to unwanted parties [54].

Poor breach notification is also an important privacy issue [73]. For example, the PIPEDA proposed a new requirement for organizations to report material data breaches to the Privacy Commissioner of Canada and to notify individuals where there is a risk of harm [64]. Unfortunately, the breach notification does not really protect a customer’s privacy. A recent survey shows that consumers who have received data breach notifications within the past year are at a much greater risk for fraud than the typical consumer [74].

## Cloud Computing Strategic Planning

When a health organization considers moving its service into the cloud, it needs strategic planning to examine the new model’s benefits and risks, assess its capabilities to achieve the goal, and identify strategies designed for its implementation. Several references are available for establishing a cloud strategic plan. For example, Marks and Lozano [75] describe the cloud computing adoption life cycle method involving 9 stages to help users begin a cloud project. These are proof of concept/pilot project, strategy and roadmap, modeling and architecture, implementation planning, implementation, expansion, integration, collaboration, and maturity. 

The Project Management Institute, a not-for-profit membership association for the project management profession, published a white paper on cloud computing that can be used as a reference for any cloud project manager. The paper provides 8 key steps for implementing cloud computing, as well as 2 case studies that support the method [76].

Stanoevska-Slabeva et al [77] also provide practical guidelines for moving traditional IT infrastructure toward clouds: initial analysis of demand and readiness for cloud computing, strategic decision to introduce cloud computing, pilot implementation, internal interconnection, inclusion of external resources, and continuous monitoring and evaluation.

The US Federal Health IT Strategic Plan [78], released in June 2008, can also be used for large government bodies to implement health cloud projects. The Plan charged the Office of the National Coordinator for Health Information Technology with a leadership role for the development and nationwide implementation of an interoperable health IT infrastructure to improve the quality and efficiency of health care. The strategic plan has 2 goals: patient-focused health care and population health, with 4 objectives under each goal. The objectives for both goals are privacy and security, interoperability, adoption, and collaborative governance. The Plan for achieving each goal is detailed through 43 strategies that describe the work needed to achieve each objective. Each strategy is associated with a milestone against which progress can be assessed and a set of illustrative actions to implement each strategy.

**Figure 2:**
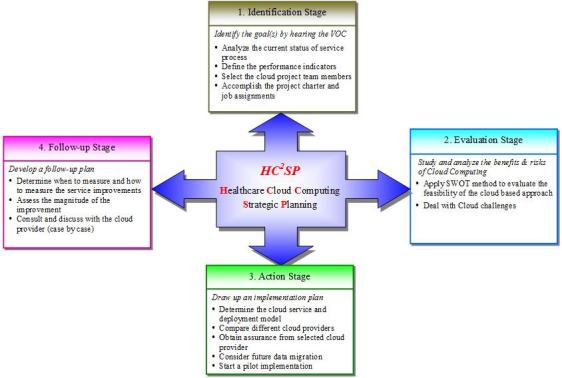
Health care cloud computing strategic planning (HC^2^SP) model (SWOT = strengths, weaknesses, opportunities, and threats; VOC = voice of customer).

Besides the above-discussed strategic planning methods, this paper, based on a study [79], proposes a health care cloud computing strategic planning (HC^2^SP) model that can be used by a health organization to determine its direction, strategy, and resource allocation to migrate from traditional health services to cloud-based services. The model includes 4 stages: identification, evaluation, action, and follow-up (see Figure 2).

### Stage 1: Identification

In this HC^2^SP model, the first stage is to analyze the current status of the health organization’s service process and identify the fundamental objective of service improvement by hearing the voice of the customer or the patients. The root causes analysis method can be applied to analyze the problems of the current service process. A typical hierarchy of causes would be expressed as follows [80]:

*Problem #1*: The process of patient admission to, or discharge from, hospital is too long. Why? There is too much unnecessary (duplicate) charting. Why? The paper-based charting system is inefficient. Why? There is lack of automated information systems such as EHR/EMR. Why? It involves a lot of up-front IT investments and maintenance.

The objective identification and its scope must be clarified so as to serve the end users (patients) more efficiently and effectively. In addition, the strategic planning team has to define health care service quality indicators and explain their purpose as well as the use of each indicator. This stage of the model provides the strategic planning team with a well-defined scope for the service problem being faced.

### Stage 2: Evaluation

The second stage of the model is to evaluate the opportunities and challenges of adopting cloud computing. ENISA [54], the Cloud Security Alliance [62], and NIST [53] have developed comprehensive guides to evaluate the benefits and risks of adopting cloud computing. A potential user can also apply a strengths, weaknesses, opportunities, and threats (SWOT) analysis to evaluate the feasibility of the cloud-based approach [81].

Furthermore, the user needs to assess methods to handle the identified issues. Many references are available for this purpose (see Table 2 [51,53,62,71,82,83]). For example, Armbrust et al [51] report 10 major obstacles for cloud computing. Each obstacle is paired with opportunities (solutions), ranging from straightforward product development to major research projects. Buyya and Ranjan [82] discuss several cloud-federated management issues, such as data transfer bottlenecks, shared logging, and federation of distributed clusters. They also provide further references to handle the discussed issues. In addition, Kuo et al [83] propose an XML-based mediator to conquer data lock-in problems.

**Table 2:** Potential solutions to the cloud computing challenges

Challenges	Resources	Solution Summary
Management and technical issues	Armbrust et al [51]	Ten solutions to handle technical, policy, and business issues
	Buyya and Ranjan [82]	Further references to handle cloud-federated management issues
	Kuo et al [83]	XML-based mediator to handle data lock-in (interoperability) problems
Security and legal issues	Cloud Security Alliance [62]	Solutions to handle cloud governance and operation issues (12 domains)
	NIST^a^ guidelines [53]	Precaution recommendations to deal with security and privacy issues
	Ward and Sipior [71]	Five strategies for handling data jurisdiction issues

^a^ National Institute of Standards and Technology.

The Cloud Security Alliance [62] describes 12 domains of concerns for cloud computing. The domains are divided into 2 broad categories: governance and operations. Solution recommendations are also provided for each domain. The NIST Guidelines on Security and Privacy in Public Cloud Computing [53] names many key cloud security and privacy issues and the corresponding precaution recommendations for organizations to follow when planning or initiating a public cloud service outsourcing arrangement. Ward and Sipior [71] focus on jurisdiction issues. They recommend 5 strategies for cloud customers to deal with jurisdiction problems. 

### Stage 3: Action

After evaluating the new computing model, the organization will be able to determine whether to adopt the service or not. If the answer is yes, it needs to draw up an implementation plan. This paper proposes a 5-step plan as follows.

#### Step 1: Determine the Cloud Service and Deployment Model

As discussed above, cloud computing can refer to several different service types (SaaS, PaaS, and IaaS) and different deployment models (private, public, community, and hybrid cloud). Each service type or deployment model has its own benefits and risks [55]. Therefore, the key considerations in contracting for different types of services or deployment models should be different.

#### Step 2: Compare Different Cloud Providers

Choosing a proper cloud provider is the most important part of the implementation plan. Different providers may offer different service models, pricing schemes, audit procedures, and privacy and security policies. The organization has to compare different offerings. Also, it needs to evaluate the provider’s reputation and performance before it signs a contract.

#### Step 3: Obtain Assurance From Selected Cloud Provider

The organization needs assurances that the selected provider will provide quality of service and follow sound privacy, security, and legal practices and regulations. The quality-of-service assurances include on-demand access, pay-per-use, rapid-elasticity, on-time troubleshooting support, and operational transparency [54]. The privacy and security assurances cover data confidentiality, integrity, availability, authenticity, authorization, and nonrepudiation. Also, the provider must assure that the data, including all of its backups, are stored only in geographic locations permitted by contract, service level agreement, and regulation.

#### Step 4: Consider Future Data Migration

The organization may have to move data and services to another provider or back to an in-house IT environment because the provider ceases business or service operations (eg, the recent discontinuation of Google Health [2]), has an unacceptable decrease in service quality, or has a contract dispute. Data portability must be considered up front as part of the plan [84].

#### Step 5: Start a Pilot Implementation

Many previous strategic planning methods suggest that an organization with no previous cloud experience start with a pilot implementation [75,77]. The pilot should be suitable for providing proof of the advantages of cloud computing for the organization.

### Stage 4: Follow-up

The last stage is to deploy the cloud computing infrastructure and develop a follow-up plan. The plan indicates when to measure and how to measure the service improvements. Reasonable targets are established beforehand, and the results of the new services are measured against the specified targets or performance indicators to assess the magnitude of the improvement [80]. If the new service condition is not satisfied, the health organization needs to review what facts influence the objective achievement. If the main cause of unsatisfied service condition is from the cloud provider, the organization will consult and discuss with the provider to improve service or may consider moving data and services to another provider or back to its in-house IT environment.

## Discussion and Conclusion

Cloud computing is a new model of computing that promises to provide more flexibility, less expense, and more efficiency in IT services to end users. It offers potential opportunities for improving EHR adoption, health care services, and research. However, as discussed above, there are still many challenges to fostering the new model in health care. Perhaps the strongest resistance to the adoption of cloud computing in health IT centers concerns data security and legal issues. Fortunately, many main providers (eg, Microsoft, Google, Amazon) have commitments to develop best policies and practices to secure customer’s data and privacy [59-61]. Some not-for-profit organizations, such as the Cloud Security Alliance and the Trusted Computing Group, have developed comprehensive guidelines, and hardware and software technologies to enable the construction of trustworthy cloud applications. Governments also foster regulations (eg, HIPAA [63] and PIPEDA [64]) to protect cloud users’ data security and privacy. In addition, most legal issues involved in cloud computing usually can be resolved through contract evaluation or negotiations [10,54].

When a health organization considers moving its service into the cloud, it needs strategic planning to examine environmental factors such as staffing, budget, technologies, organizational culture, and government regulations that may affect it, assess its capabilities to achieve the goal, and identify strategies designed to move forward. This paper provides useful strategic planning references for potential users to start cloud projects. Also a new model called HC^2^SP is proposed that could be applied by a health organization to determine its direction, strategy, and resource allocation to move to the cloud paradigm. The model includes 4 stages: identification, evaluation, action, and follow-up. At the first stage, the organization analyzes the current status of the service process and identifies the fundamental service objective. Stage 2 is to evaluate the opportunities and challenges of adopting cloud computing. By using the SWOT analysis, the organization can determine the internal strength and weakness factors as well as the external opportunity and threat factors of adopting the new model. Some potential solutions to handle cloud issues have been also provided. Then, in stage 3, the organization draws up a cloud computing implementation plan. The author suggests that this should include at least the following: determine the cloud service and deployment model, compare different cloud providers, obtain assurance from the selected cloud provider, consider future data migration, and start a pilot implementation. The last stage is to deploy the cloud computing infrastructure and develop a follow-up plan to measure the health care service improvements. 

As the Chief Executive Officer of a cloud IT company commented [85]:

If you woke up this morning and read in The Wall Street Journal that, say, Overstock.com has stopped using UPS and FedEx and the U.S. mail, and had bought fleets of trucks and started leasing airport hubs and delivering products themselves, you would say they were out of their minds. Why is that much more insane than a health care company spending $2 billion a year on (traditional) information technology?

Cloud computing presents a compelling opportunity for consumers of IT and producers of information services [86,87]. Gartner Research also found that cloud computing ranked as the top technical priority of chief information officers in 2011 [88]. However, adopting cloud computing is a complex process involving many factors. It needs rigorous evaluation before introducing the new computing model to an organization. This paper focuses on 4 aspects of evaluation and strategic planning, which will assist health organizations in determining whether (or how) to migrate from traditional to cloud-based health services.

## Abbreviations

Amazon S3: Amazon Simple Storage Service
AWS: Amazon Web Services
CPU: central processing unit
EC2: Elastic Compute Cloud
EHR: electronic health record
ENISA: European Network and Information Security Agency
HC2SP: health care cloud computing strategic planning
HIPAA: Health Insurance Portability and Accountability Act
IaaS: infrastructure as a service
IT: information technology
NIST: National Institute of Standards and Technology
PaaS: platform as a service
PATRIOT: Uniting and Strengthening America by Providing Appropriate Tools Required to Intercept and Obstruct Terrorism
PIPEDA: Personal Information Protection and Electronic Documents Act
SaaS: software as a service
SSAHA: sequence search and alignment by hashing
SWOT: strengths, weaknesses, opportunities, and threats
